# Combining Passive Sampling and Dosing to Unravel the Contribution of Hydrophobic Organic Contaminants to Sediment Ecotoxicity

**DOI:** 10.1021/acs.est.3c07807

**Published:** 2023-12-28

**Authors:** Nienke Wieringa, Steven T. J. Droge, Thomas L. Ter Laak, Aishwarya A. K. Nair, Kelsey Walker, Piet F. M. Verdonschot, Michiel H. S. Kraak

**Affiliations:** † Department of Freshwater and Marine Ecology (FAME), Institute for Biodiversity and Ecosystem Dynamics (IBED), University of Amsterdam, Science Park 904, 1098 XH Amsterdam, The Netherlands; ‡ Wageningen Environmental Research, Wageningen University and Research, P.O. Box 47, 6700 AA Wageningen, The Netherlands; § KWR Water Research Institute, Groningenhaven 7, 3433 PE Nieuwegein, The Netherlands

**Keywords:** passive sampling, passive dosing, sediment contamination, bioassay, Chironomus riparius, isolating toxic pressure, organic contaminants

## Abstract

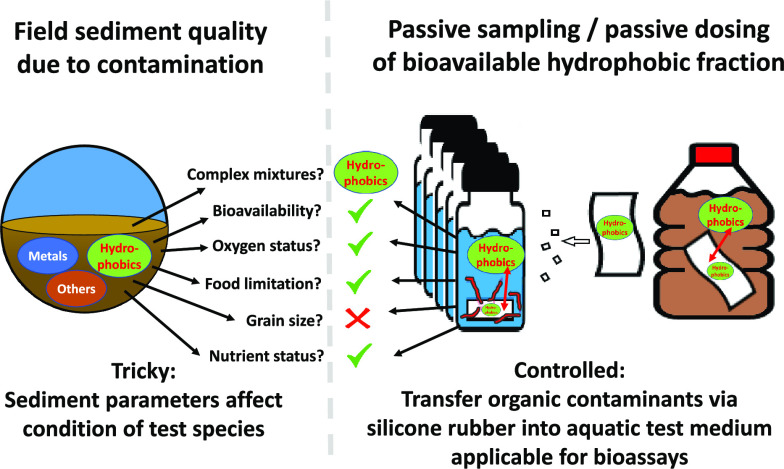

Contaminated sediments are ubiquitous repositories of pollutants and cause substantial environmental risks. Results of sediment bioassays remain difficult to interpret, however, as observed effects may be caused by a variety of (un)known stressors. This study aimed therefore to isolate the effects of hydrophobic organic contaminants from other (non)chemical stressors present in contaminated sediments, by employing a newly developed passive sampling–passive dosing (PSPD) test. The results showed that equilibrium partitioning between pesticides or polyaromatic hydrocarbons (PAHs) in contaminated sediments and a silicone rubber (SR) passive sampler was achieved after 1–3 days. Chlorpyrifos concentrations in pore water of spiked sediment matched very well with concentrations released from the SR into an aqueous test medium, showing that SR can serve as a passive dosing device. Subjecting the 96 h PSPD laboratory bioassay with nonbiting midge (*Chironomus riparius*) larvae to field-collected sediments showed that at two locations, concentrations of the hydrophobic organic contaminant mixtures were high enough to affect the test organisms. In conclusion, the developed PSPD test was able to isolate the effects of hydrophobic organic contaminants and provides a promising simplified building block for a suite of PSPD tests that after further validation could be used to unravel the contribution of hydrophobic organic chemicals to sediment ecotoxicity.

## Abstract 2

To isolate the effects of hydrophobic organic toxicants in contaminated sediments from other (non)chemical stressors present in multistress environments, a passive sampling–passive dosing (PSPD) test with larvae of *Chironomus riparius* was developed and successfully employed.

## 1 Introduction

Contaminated sediments are ubiquitous repositories of pollutants, harboring toxic chemicals at concentrations many times higher than in the overlaying water.^1^ Consequently, sediments may serve as a continuous source of contaminants to the overlying water,^2^ which adversely affect aquatic ecosystems, causing substantial environmental risks.^3−6^ Nonetheless, contaminated sediments are largely overlooked in water quality assessments performed according to the European Union Water Framework Directive (EU-WFD),^5,7^ which requires member states to monitor 45 priority substances in the water, but not in the sediment.^8^ If performed at all, chemical analysis of a limited number of contaminants does not accurately characterize the complex contaminant mixtures present in polluted sediments^9^ nor the actual exposure of organisms, expressed as the freely dissolved concentration, the chemical activity, or the bioaccessibility. Alternatively, bioassays may be employed as they respond to all known and unknown bioavailable contaminants present.^10^ Yet, bioassay results are sometimes difficult to interpret as the observed adverse effects may be caused by a variety of (un)known stressors.^6^ The presence of high nutrient levels and differences in sediment composition of contaminated sediments may either mask or exaggerate the potential adverse effects of the chemical contaminants.^6,11,12^ Also, the choice of the test organism or end point may lead to differential outcomes of the bioassays, as different organisms and end points exhibit specific sensitivities to the wide variety of compounds present in the complex contaminant mixtures.^13^

Mixtures of sediment associated contaminants mostly comprise chemicals that are strongly sorbed, including hydrophobic polyaromatic hydrocarbons (PAHs), dioxins, flame retardants and certain pesticides, personal care product ingredients, and pharmaceuticals. In addition, sediments may also retain more polar organic cations, such as various illicit drugs, pharmaceuticals, and fabric softeners,^14−17^ and some amphiphilic compounds such as per- and polyfluoroalkyl substances (PFAS).^18^ The relative contribution of each of these contaminant categories to the outcome of sediment bioassays is difficult to pinpoint,^19−21^ which requires to tease out all groups of contaminants present and to isolate category by category. Here, we argue that this may be achieved by employing passive samplers, as these are able to extract and transfer chemical contamination from field-contaminated sediments into controlled water-only (eco)toxicity tests, while rendering confounding sediment-related factors negligible.^22−26^

Equilibrium passive sampling with rubbery polymer phases is a promising approach to determine the bioavailable fraction of neutral organic contaminants from aqueous environments and from sediments.^27−29^ A thin polymer sheet, such as silicone rubber (SR), is expected to equilibrate with the freely dissolved concentration of neutral organic contaminants,^30^ which is a direct metric for the toxic potential of soluble organic contaminants to aquatic organisms.^31^ The polymer sampler equilibrated with contaminated sediment can subsequently be used as a dosing phase to release the accumulated compounds into an aqueous test solution, which will mimic the original composition and concentration profile of the sediment pore water.^25,32−34^ The compounds present in the passive doser will equilibrate with the aqueous test solution according to the polymer–water partition coefficient, similar to the chemical profile in the pore water of the sediment phase, as long as the sediment phase is not substantially depleted by the deployment of the polymer as a passive sampler and the sampler itself is not substantially depleted during its deployment as a passive doser. The passively dosed water would thus contain the polymer-transferable organic contaminants at the same chemical activity as the sediment pore water and thus serve as a convenient test medium for investigating sediment ecotoxicity while avoiding the potential confounding influence of other contaminant or noncontaminant stressors.^35^ Experimental verification of this approach may pave the way toward a broader application of simplified but representative aqueous bioassays for sediment quality assessment. This study aimed therefore to isolate the effects of hydrophobic organic toxicants in contaminated sediments from other (non)chemical stressors present in a multistress environment by employing a newly developed passive sampling–passive dosing (PSPD) test. To this end, a PSPD test was developed to sample hydrophobic organic toxicants from contaminated sediments and to dose these toxicants into an aqueous medium. Next, laboratory water-only bioassays were performed with the developed PSPD test, assessing the effect of a range of contaminated sediments on larvae of the nonbiting midge *Chironomus riparius*.

## 2 Materials and Methods

### 2.1 Outline of the Study

**Figure 1:**
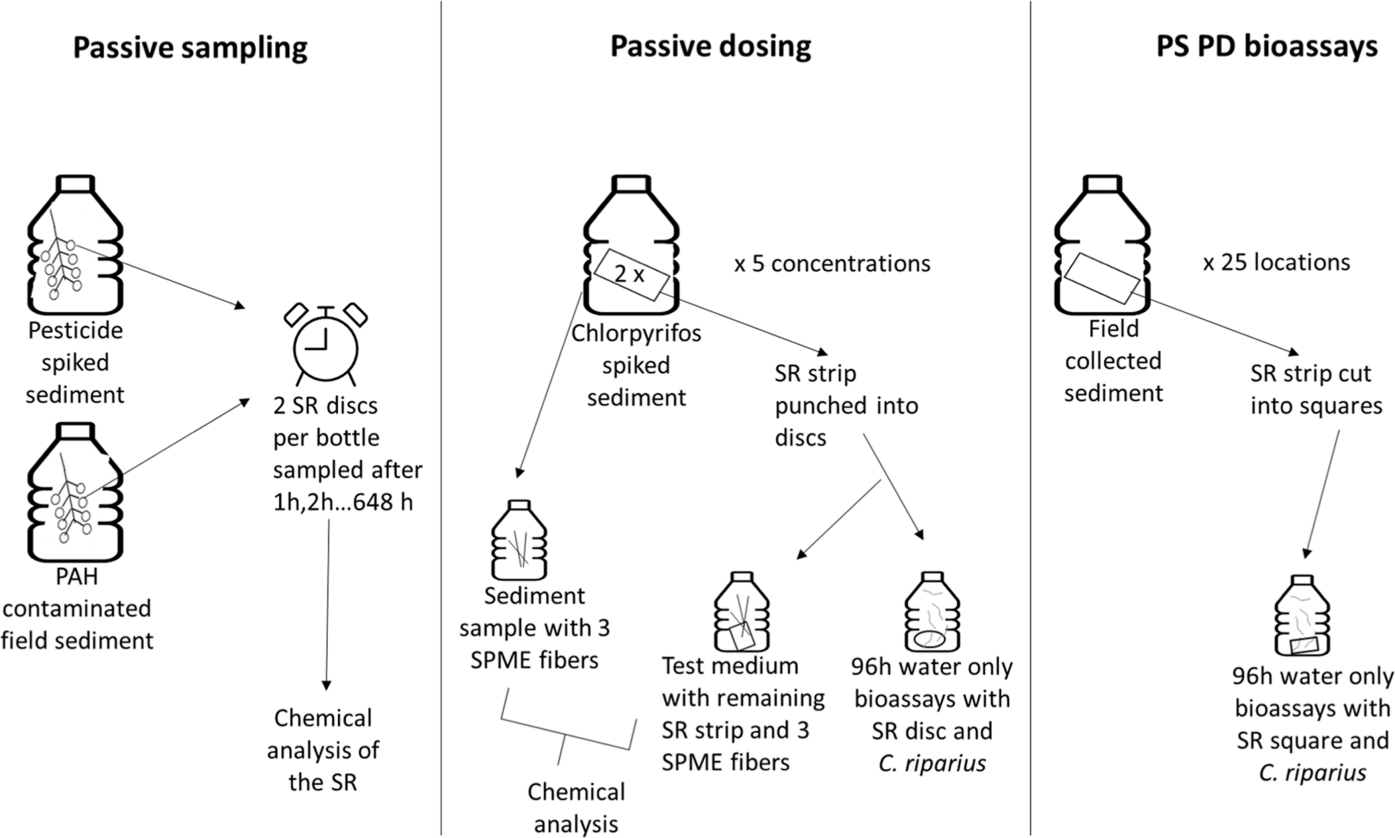
Graphical outline of the study for the passive sampling (PS), passive dosing (PD), and PSPD bioassays. SR = silicone rubber and SPME = solid-phase microextraction.

The present study followed a stepwise approach. First, the passive sampling (PS) of organic compounds from contaminated sediments was verified. To this end, the required passive sampling equilibration time of 0.5 mm thick sheets of silicone rubber (SR) for organic compounds in a 1:1 sediment/water slurry was determined. We tested this for a reference sediment spiked with seven pesticides with a broad range in hydrophobicity and a field sediment with historic polycyclic aromatic hydrocarbon (PAH) contamination (Figure 1, left panel and Table S1). Second, we verified the passive dosing (PD) of an aqueous solution by SR equilibrated with reference sediment spiked with different concentrations of the insecticide chlorpyrifos. To this end, we compared the chlorpyrifos concentrations in the pore water of the spiked sediment with those released from the SR into an aqueous solution (Figure 1, middle panel and Table S1). The transfer of the toxicant, and thus the toxic potential, from the sediment slurry into a 96 h water-only PSPD bioassay was evaluated by dosing aqueous solutions by SR equilibrated with reference sediment spiked with different concentrations of the insecticide chlorpyrifos to which larvae of the nonbiting midge *C. riparius* were exposed (Figure 1, middle panel and Table S1). After verification of the PS and PD steps, the 96 h PSPD laboratory bioassay was subjected to a wide range of field-collected sediments (Figure 1, right panel and Table S1).

### 2.2 Passive Sampling

#### 2.2.1 Sediment Collection and Preparation

Reference sediment was sampled from a relatively uncontaminated shallow ditch^36^ at Amsterdam Science Park (see Table S2) using an Ekman grab sampler. To eliminate any indigenous fauna, the sediment was sieved over a 2 mm sieve and stored for 1 week at −20 °C. Three days before the start of the passive sampling experiment, a batch of the reference sediment was thawed and air-dried at 70 °C over 48 h. Approximately 375 g of wet sediment was spiked with a pesticide mixture dissolved in HPLC grade acetone (J.T. Baker, Deventer, The Netherlands), by soaking the sediment in 200 mL of spike solution in a 2 cm deep aluminum foil tray. The pesticide mixture consisted of the insecticides propoxur, carbofuran, pirimicarb, quinalphos, and chlorpyrifos (all acetylcholinesterase inhibitors); the insecticide fipronil (GABA-gated chloride channel antagonist); and the herbicide linuron (photosystem II inhibitor) (see Table S3). Each pesticide was present in the mixture at one specific concentration (Table S4), based on approximately 10 times the 50% lethal concentration for aquatic invertebrates (LC_50_),^37^ the reported soil organic carbon sorption coefficients (*K*_oc_),^37^ and an estimated 5% organic carbon (*f*_oc_) content of the sediment, obtained from our previous study:^36^
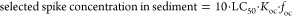
1

After the acetone was evaporated overnight in a fume hood, the spiked sediment was carefully mixed with 800 g of wet weight of untreated sediment and put into a 1 L glass bottle with a Teflon-lined screw cap (Duran, Mainz, Germany). A total of 300 mL of demineralized water was added to the wet sediment in each bottle to create a slurry, which was then mixed on a rolling bank (Bellco, Vineland, NJ) for 4 weeks and left at room temperature for another 8 weeks (100 days in total since spiking).

Contaminated field sediment samples with historic PAH pollution were used to monitor the uptake of phenanthrene and pyrene by the SR. To this end, sediment samples were taken with a core sampler (UWITEC, Mondsee, Austria) in two Amsterdam canals (see Table S2) and stored frozen at −20 °C. After thawing, ∼500 g of sediment was taken, mixed for 1 week on a rolling bank in a 1 L glass bottle with 0.5 L of deminerilized water, and then stored for 100 days at room temperature.

#### 2.2.2 SR Preparation and Passive Sampling

Silicone rubbers (SRs) consisting of polydimethylsiloxane (PDMS) of 500 μm thickness (Altec, St Austell, United Kingdom) were cut into discs with a 13 mm diameter circular punch (132 mm^2^, 0.10 g). SR discs were cleaned by immersion in HPLC grade ethyl acetate (J.T. Baker, Deventer, The Netherlands) for 24 h, after which they were placed in HPLC grade methanol (J.T. Baker) for 1 h, air-dried, and then rinsed four times with demineralized water. For both the pesticide-spiked reference sediment and PAH-contaminated field sediment, 26 SR discs were prepared. To submerge the prepared SR discs in the sediment slurries and to facilitate sampling at different exposure times, a piece of galvanized wire (1.3 mm diameter, GAH Alberts, Herscheid, Germany) was pierced through each disc and twisted after each disc to hold them separated in place along the wire. Next, the bottles were placed horizontally on a roller bank at 20 rpm. Duplicate SR discs were sampled from each bottle over a period of 2 weeks after 1, 2, 4, 7, 24, 48, 72, 96, 120, 144, and 312 h of exposure.

#### 2.2.3 Chemical Analysis of the SR Discs

For the extraction of the pesticides and the PAHs, the SR discs were placed individually in HPLC vials with 1.5 mL of 1:1 methanol/acetonitrile (HPLC grade, J.T. Baker) for 24 h on a Stuart SRT9 roller mixer (Cole-Parmer, Stone, U.K.). LC-MS/MS detection of the pesticides was performed on a Prominence UFLC-XR (Shimadzu, Kyoto, Japan), coupled to a tandem mass spectrometer (QTRAP 4000, Applied Biosystems) using a 100 mm × 2.1 mm (2.7 μm Express C18) Ascentis column (Supelco, Darmstadt, Germany) (see Section S1 and Table S5).

For PAH analysis, the SR extracts taken on the first day were diluted 50 times, while the other extracts were diluted 100 times with 1:1 methanol/acetonitrile. HPLC fluorescence detection was performed on a Prominence UFLC-XR (Shimadzu) system using a 100 mm × 3 mm (2.60 μm XB-C18) Kinetex column (Phenomenex, Torrance, CA) (Section S2 and Table S6).

A first-order exponential uptake curve was fitted through the measured contaminant concentrations in the SR discs (*C*_SR_, in mg/kg) collected after different exposure times to the sediment slurry, using GraphPad Prism (GraphPad Software Inc., version 9.1.3, San Diego, CA) applying the formula:
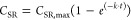
2in which *C*_SR,max_ is the equilibrium concentration between the sediment and the SR, and *k* (day^–1^) is the uptake rate constant.

The time it took to reach 95% of the equilibrium concentration for the individual compounds in the SR (*t*_95,SR_) was then calculated according to
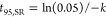
3

### 2.3 Passive Dosing

#### 2.3.1 Sediment Collection and Preparation

To evaluate if the SRs could serve as passive dosing devices to deliver the accumulated contaminants to an aqueous phase, the same reference sediment was used to create five batches of chlorpyrifos-spiked sediment using the method described above (see Section 2.2.1). The spike solution was diluted five times with acetone to create five concentrations of which the middle concentration was intended to reach a chlorpyrifos concentration of 1 μg/L in the aqueous phase, as previous research showed that LC_50_ values of chlorpyrifos for chironomids ranged from 70 to 825 ng/L for 2–10 days ecotoxicity tests.^38−44^ Spike solution concentrations were based on the estimation that the sediment had an *f*_oc_ of 0.05 and a *K*_oc_ of 9930 L/kg for chlorpyrifos. Nominal concentrations of the other four spiked chlorpyrifos concentrations in the sediment can be found in Table S7. An acetone control was included, as well. The bottles prepared with the slurries (750 g of air-dried sediment and 750 mL of water) were placed on a roller bank for 7 days, after which the SR was added.

#### 2.3.2 SR Preparation and Passive Sampling

Silicone rubber was cut into strips of 6 × 3 cm^2^ (approximately 1.4 g). Per bottle, two SR strips were added, amounting to a SR to sediment organic carbon ratio of 1:12.5. The strips were submerged in the sediment slurries by attachment to the middle section of a galvanized wire coil, which was placed between the bottle cap and bottom. The bottles were placed for 27 days on a roller bank at room temperature at 20 rpm. After 27 days, the SR strips were cleaned by rinsing with Milli-Q water (>18.2 MΩ·cm^–1^) prepared with a Milli-Q system (MilliPore, Amsterdam, The Netherlands) and dried on tissue. The circle punch was used to cut out eight 0.1 g discs per strip, which were wrapped in aluminum foil and stored at −20 °C until use in the 96 h PDPS bioassay (see Section 2.3.5). Per bottle, a part of one of the remaining SR strips was weighed and used to determine the chlorpyrifos concentration released by passive dosing from the SR into the aqueous solution, as described below.

#### 2.3.3 Comparison of the Chlorpyrifos Concentrations Released from the SR into the Aqueous Test Medium with Those in the Pore Water of the Spiked Sediment

The chlorpyrifos concentrations released from the SR into the aqueous test medium were compared to those in the pore water of the spiked sediment. To this end, polyacrylate-coated solid-phase microextraction (SPME) fiber (Polymicro Technologies, Phoenix, AZ) was purchased as a single strand of 200 m length, consisting of glass fiber with an internal diameter of 108 μm and a 34.5 μm polyacrylate coating with a volume of 15.4 μL/m of fiber. Bundles of 20 SPME fibers were wrapped in aluminum foil and cut into pieces of 40 mm, resulting in a reproducible polyacrylate volume of 0.62 μL. SPME fibers were precleaned by immersion in methanol for 1 h, after which the fibers were stored in Milli-Q water.

To measure the chlorpyrifos concentrations released from the SR into the aqueous test medium, triplicate SPME fibers were deployed in a 10 mL Dutch standard water (DSW) solution^46^ that had been dosed for 7 days with a part of the remaining SR strip (∼0.3 g) after cutting out the discs. This was done for each original chlorpyrifos concentration spiked to the reference sediment and for the acetone control. The vials with SPME fibers were agitated for 7 days on a roller mixer (20 rpm) at 20 °C to ensure equilibration between the SPME fiber and the chlorpyrifos-containing aqueous solution.

To measure the chlorpyrifos concentrations in the pore water of the spiked sediment, SPME fibers were deployed in sediment subsamples collected 1 day after taking out the SR strips, i.e., 28 days after spiking. From each bottle with chlorpyrifos-spiked sediment, 4 mL of wet sediment was put into a 10 mL vial. Then, 4 mL of demineralized water was added to create a slurry, 1 mL of formaldehyde solution (37%, J.T. Baker) was added to prevent biodegradation of chlorpyrifos, and triplicate SPME fibers were added as passive samplers. The vials with SPME fibers were agitated for 7 days on a roller mixer (20 rpm) at 20 ± 1 °C to ensure equilibration between the SPME fiber and the chlorpyrifos-containing sediment.

#### 2.3.4 Chemical Analysis of the Passive Sampler Extracts

SPME fibers were collected from the vials with solvent-cleaned stainless-steel tweezers and wiped clean with a Milli-Q wetted tissue. Each SPME fiber was cut into 1 cm pieces that were collected into a 300 μL insert in an HPLC vial. Chemicals were desorbed from the polyacrylate coating with 200 μL of acetonitrile during at least 24 h on a roller mixer. After extraction, 50 μL of Milli-Q water was added to the vials before analysis by LC-MS/MS. The analytical methods and the respective limits of detection and limits of quantification can be found in Section S1 and Table S5. The freely dissolved chlorpyrifos concentrations in the pore water of the spiked sediment and in the aqueous solution dosed with the chlorpyrifos-containing SR were obtained by dividing the measured SPME polyacrylate concentration by the polyacrylate–water partition coefficient (*K*_pa–w_) reported as 1.5 × 10^5^ by Magdic et al.^45^

#### 2.3.5 PSPD Bioassays with the Chlorpyrifos-Spiked Sediment

The 96 h PSPD bioassays were performed with first instar larvae (<24 h) of the nonbiting midge *C. riparius* taken from the University of Amsterdam in-house laboratory culture. The culture was kept in several 20 L aquaria containing quartz sand overlaid with DSW and was fed a mixture of Trouvit (Trouw, Fontaine-les-Vervins, France) and Tetra Phyll (Tetra Werke, Melle, Germany) in a ratio of 20:1. This mixture was also used as food in the 96 h PSPD bioassays. The constantly aerated cultures were kept at 20 ± 1 °C, 65% humidity, and a 16:8 h light–dark photoperiod.

The first instar *C. riparius* larvae were exposed for 96 h to aqueous solutions that had been dosed for 7 days with the punched SR discs. The PSPD bioassay consisted of five chlorpyrifos SR dosing levels, with five replicates per treatment. Each experimental replicate consisted of a 10 mL glass vial with 1.8 mL of DSW and 0.2 mL of food solution (17.5 mg/mL) and one chlorpyrifos-containing SR disc equilibrated for 7 days with the chlorpyrifos-containing sediment. The control treatment (*n* = 5) consisted of vials containing cleaned SR discs and food. Before the beginning of the PSPD bioassay, 1 mL of hyperoxidized DSW was added to the glass vials to ensure oxygen-rich conditions. Next, five first-stage instar larvae of *C. riparius* were added to each vial, after which the vial was closed. The PSPD bioassay was conducted in a climate room at 20 °C with 65% humidity and a 16:8 h light–dark photoperiod. Larval survival was recorded after 96 h of exposure. A concentration–response relationship was constructed by plotting the survival data against the SPME-derived chlorpyrifos concentrations in the SR-dosed aqueous test solution. From this concentration–response relationship, the LC_50_ of chlorpyrifos for *C. riparius* was derived in GraphPad Prism v 9.3 according to the following formula:
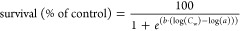
4in which relative weighting by 1/*Y*^2^ was applied, and *a* and *b* fitting parameters representing the LC_50_ and Hill slope, respectively.

### 2.4 PSPD Bioassays with Field Sediments

#### 2.4.1 Sediment Collection and Preparation

The PSPD laboratory bioassays were conducted with sediment from 25 water bodies, with varying degrees of multistress.^6,36^ Sampling was conducted in 2017 (4 sample sites) and 2018 (21 sample sites) in The Netherlands. Sediment samples were collected from nature reserves (N1–N5), water bodies receiving wastewater treatment plant (WWTP) effluent (W1–W6), agricultural areas (A1–A7), an urban area (U1), and from locations with multiple contaminant influences, originating from varying surrounding land use, labled mixed locations (M1–M6) (see Table S8). To collect the sediment cores, a sediment core sampler (UWITEC, Mondsee, Austria) was used loaded with an acrylic tube (*l*: 60 cm, *d*: 6 cm). In the laboratory, the top 5.5 cm of each sediment core was transferred into a small acrylic tube (*l*: 15 cm, *d*: 6 cm) using a sediment core cutter (UWITEC) and stored at −20 °C.

To prepare the sediments for the PSPD bioassay, per sample site, ∼750 g of defrosted sediment was poured into a 1 L glass bottle, and demineralized water was added until a volume of 1 L sediment–water slurry was reached. Contaminant-free artificial sediment was included in the experimental setup as a laboratory reference. Artificial sediment was prepared in a batch of 500 g according to OECD guideline 218^47^ with slight modifications^46^ containing 270 mg of food (a mixture of Trouvit and Tetra Phyll in a ratio of 20:1) and sterilized by autoclaving and homogenized in a glass bottle on a roller bank at 20 rpm for >24 h.

#### 2.4.2 SR Preparation and Passive Sampling

SR was cut into strips of 10 cm × 2 cm and cleaned thoroughly by placing them in ethyl acetate for 24 h, rinsing them with acetonitrile and subsequently air-drying them, rinsing them with demineralized water four times, and air-drying them again. To submerge the prepared SR strips in the sediment slurries, a galvanized wire (approximately 20 cm) was used to wrap around the prepared SR strips before placing them in the jars containing the sediment slurries. The jars were put on a roller bank for a minimum of 7 days at 1.4 rpm. Next, the strips were collected from the sediment, cleaned with a wet tissue, cut into squares of 1 cm^2^ (0.08 g), wrapped in aluminum foil, and stored at −20 °C until the start of the 96 h PSPD bioassays.

Sediments collected in 2017 (*n* = 4) were defrosted in May 2020, prepared, and used for passive sampling. Subsequently, SPME strips were stored for a month at −20 °C and then used for the 96 h passive dosing bioassays. Sediments collected in 2018 (*n* = 21) were defrosted, prepared, and used for passive sampling in May 2019 after which the PDMS strips were stored at −20 °C until the start of the 96 h PSPD bioassays in May 2020.

#### 2.4.3 Passive Dosing

24 h before the start of the bioassays, the aluminum-foil-wrapped SR pieces were transferred from the −20 °C storage into a refrigerator (4 °C). Five replicates were prepared per sediment sampling location and for the artificial sediment. Each experimental replicate consisted of a single SR piece of 1 cm^2^ placed in a 5 mL glass vial along with 2 mL of oxidized DSW and 10 μL (=17.5 mg/mL) of food solution (see Section 2.4.5), after which the vial was closed. The control treatment (*n* = 5) consisted of vials containing cleaned SR pieces and food. To allow sufficient time for equilibrium partitioning of the compounds between the SR passive dosing material, water, and food, all vials were stored in a refrigerator (4 °C) for 24 h. Next, the vials were taken out of the refrigerator and placed in a climate room to reach room temperature for >2h.

#### 2.4.4 PSPD Bioassays with Field Sediments

Five first instar *C. riparius* larvae (<24 h) were pipetted into each individual replicate vial, which was then closed and placed for 96 h in a climate room (20 ± 1°C, 65% humidity, and a 16:8 h light/dark photoperiod). The end points of the PSPD bioassays with field sediments were larval survival and growth after 96 h of exposure to the SR-dosed aqueous solutions. To obtain the initial larval length at the start of the bioassays, 10 randomly selected first instar *C. riparius* larvae (<24 h) were photographed with a Leica microscope (Leica, Wetzlar, Germany) at 1.6× magnification, from which their length was measured (Infinity Analyze software).

At the end of the 96 h PSPD bioassay, the vials were taken out of the climate room and individually decanted into an hourglass. Larval survival was recorded by counting the number of living individuals. Individual larval length was determined, and subsequently, individual larval growth was calculated by subtracting the average initial length from the final individual length.

#### 2.4.5 Bioassay Data Analysis

According to the Gagliardi outlier test,^48^ all organisms that displayed growth 1.5 times above and below the upper and lower quartile were excluded from further analysis. The growth of the larvae was normalized against the mean growth of the larvae in the control. For each location, five replicates containing five individual test organisms were tested. To correct for nested data and to prevent pseudoreplication, the mean normalized growth for each replicate was determined and used for further analysis. To determine if there were significant differences in growth between the test locations and the control, an ANOVA was used, followed by a Dunnett test. Statistical analysis was performed by using R Studio software (version 4.2).

## 3 Results

### 3.1 Passive Sampling

**Figure 2:**
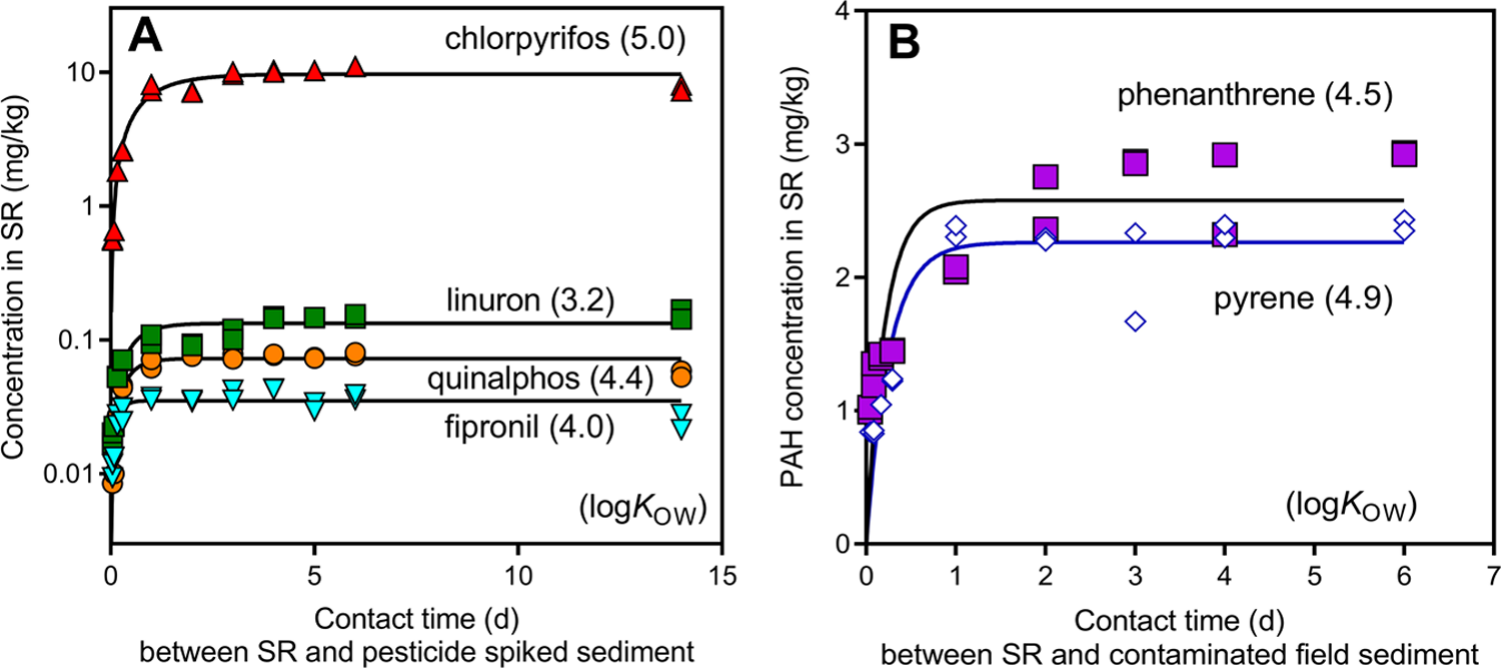
Pesticide (A) and PAH (B) concentrations in silicone rubbers (SR) (*n* = 2) after different contact times (days) with (A) sediment spiked with different pesticides and (B) contaminated field sediments. The log *K*_OW_ is given in parentheses for each chemical. The fitted lines represent a first-order exponential uptake curve. Note that panel (A) has a logarithmic *Y* axis.

The 95% equilibrium partitioning time (t_95,SR_) for the uptake of all spiked organic compounds by SR from the sediment was well reached within the 1st week of mixing on the roller bank (Figure 2 and Table S9). Chlorpyrifos, the most hydrophobic pesticide (log *K*_OW_ 5.0) present in the mixture, showed an uptake rate constant *k* of 1.2 day^–1^, resulting in a *t*_95,SR_ of 2.5 days (Figure 2a). The *t*_95,SR_ was 1.0 days for quinalphos (log *K*_OW_ 4.4), 0.4 days for fipronil (log *K*_OW_ 4.0), and 1.4 days for linuron (log *K*_OW_ 3.2). For the less hydrophobic pesticides propoxur (log *K*_OW_ 1.5), pirimicarb (log *K*_OW_ 1.7), and carbofuran (log *K*_OW_ 2.3), 1 h lead to concentration in the SR close to the equilibrium concentration (Figure S1).

The second part of the experiment was performed with PAH-contaminated field sediment and resulted in a pyrene (log *K*_OW_ 4.9) uptake rate constant *k* of 4.0 day^–1^, with a corresponding *t*_95,SR_ of 0.8 days (Figure 2b). For phenanthrene (log *K*_OW_ 4.5), an uptake rate constant *k* of 4.9 days^–1^ indicated a *t*_95,SR_ of 0.6 days.

The results of the first experiment thus showed that equilibrium partitioning of the SR with the seven pesticides in the 100 days aged spiked sediment, and PAHs in the contaminated field sediment, was achieved after 3 days of mixing on a roller device.

### 3.2 Passive Dosing

**Figure 3:**
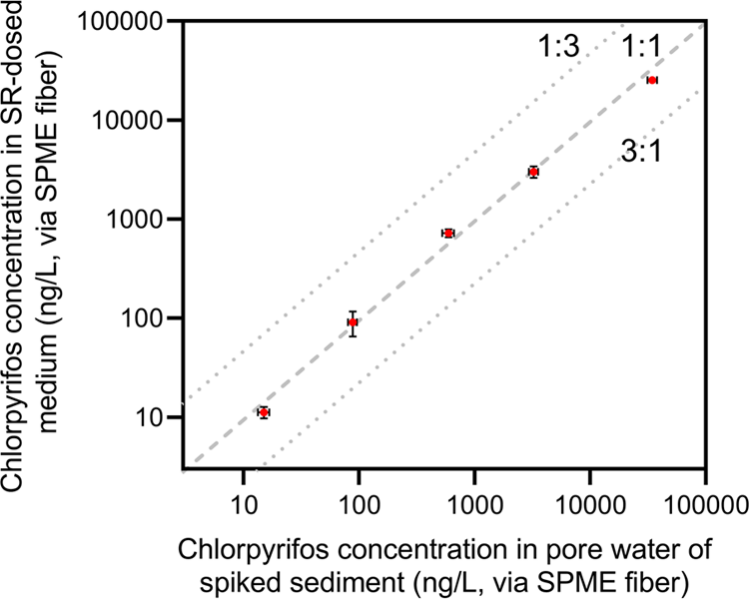
Chlorpyrifos concentration in SR-dosed medium expressed as ng/L polyacrylate in SPME fibers exposed for 7 days to this medium plotted against the chlorpyrifos concentration in 28 days old spiked sediment expressed as ng/L polyacrylate in SPME fibers exposed for 7 days to this sediment. The dashed line represents the 1:1 line, while the dotted lines include the 1:3 to the 3:1 area.

SPME fibers were used to independently measure the chlorpyrifos concentrations released from the SR into the aqueous test medium, as well as the chlorpyrifos concentrations in the pore water of the spiked sediment. As shown in Figure 3, this comparison revealed that the chlorpyrifos concentrations in the SPME fibers in the passive dosing test matched very well with the chlorpyrifos concentrations in the SPME fibers exposed to the sediment slurries (for data see Table S10). The measured freely dissolved chlorpyrifos concentrations predicted using equilibrium partitioning (EqP) between sediment and pore water were, however, higher than observed, and the predicted to measured ratio increased as the sediment spiking concentration decreased (Table S11a). Hence, the apparent silicone rubber to polyacrylate partition coefficient increased as the sediment spiking concentration decreased (Table S11b). Nonetheless, the results of this experiment show that SR can serve as a passive doser to transfer the chemical activity of chlorpyrifos and potentially other sediment-accumulated contaminants with a wide spectrum of SR–water partition coefficients, after shaking the SR for 7 days with an aqueous test medium.

**Figure 4:**
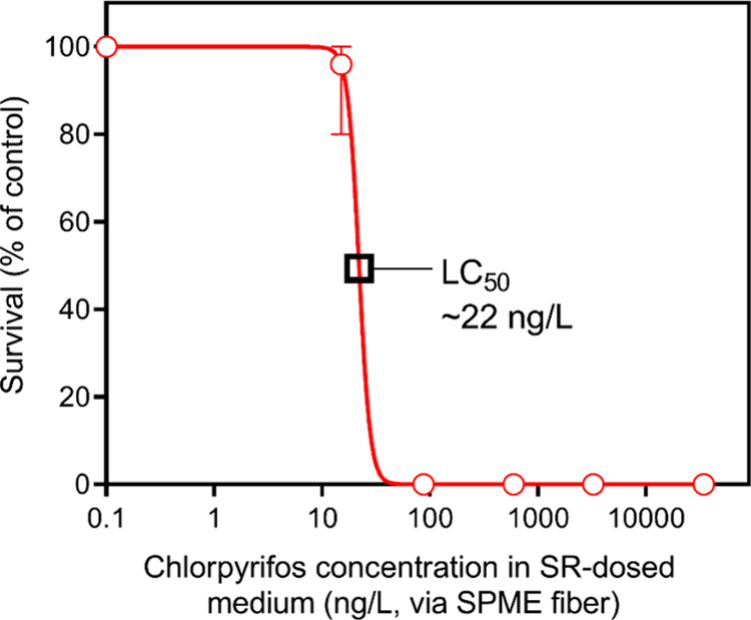
Survival (% of control) of *C. riparius* larvae after 96 h of exposure to SR-dosed medium, expressed as nanograms of chlorpyrifos/L polyacrylate in SPME fibers exposed for 96 h to this medium. Black squares indicate the LC_50_.

To study the possible use of SRs in a PSPD bioassay, the responses of first instar *C. riparius* larvae to the five passive dosing levels of chlorpyrifos were evaluated after 96 h of exposure. The solvent control resulted in a 100% survival in all replicates. The obtained concentration–effect relationship for survival (Figure 4) was based on the chlorpyrifos concentration in the test medium measured by means of SPME fibers. The four highest test concentrations all induced complete mortality of the *C. riparius* larvae, while at the lowest test concentration, four of the five replicates showed 100% survival. The calculated LC_50_ for *C. riparius* exposed to the freely dissolved chlorpyrifos in the test medium was 22 ng/L. The results of this experiment thus showed that SR can transfer the toxic potential of sediment contaminants into an aqueous phase and can serve as a passive doser in a bioassay with first instar *C. riparius* larvae.

### 3.3 PSPD Bioassay with Field Sediments

The 96 h PSPD laboratory bioassay was subjected to a wide range of field sediments. Control survival of the *C. riparius* larvae in the 96 h PSPD aqueous bioassays was 100% ± 0.0 (mean ± SE). Larval survival in the test solutions equilibrated with SR exposed to field sediments was at least 88%, and no significant differences (*p* > 0.05) in survival compared to the corresponding control were observed (Figure S2).

**Figure 5:**
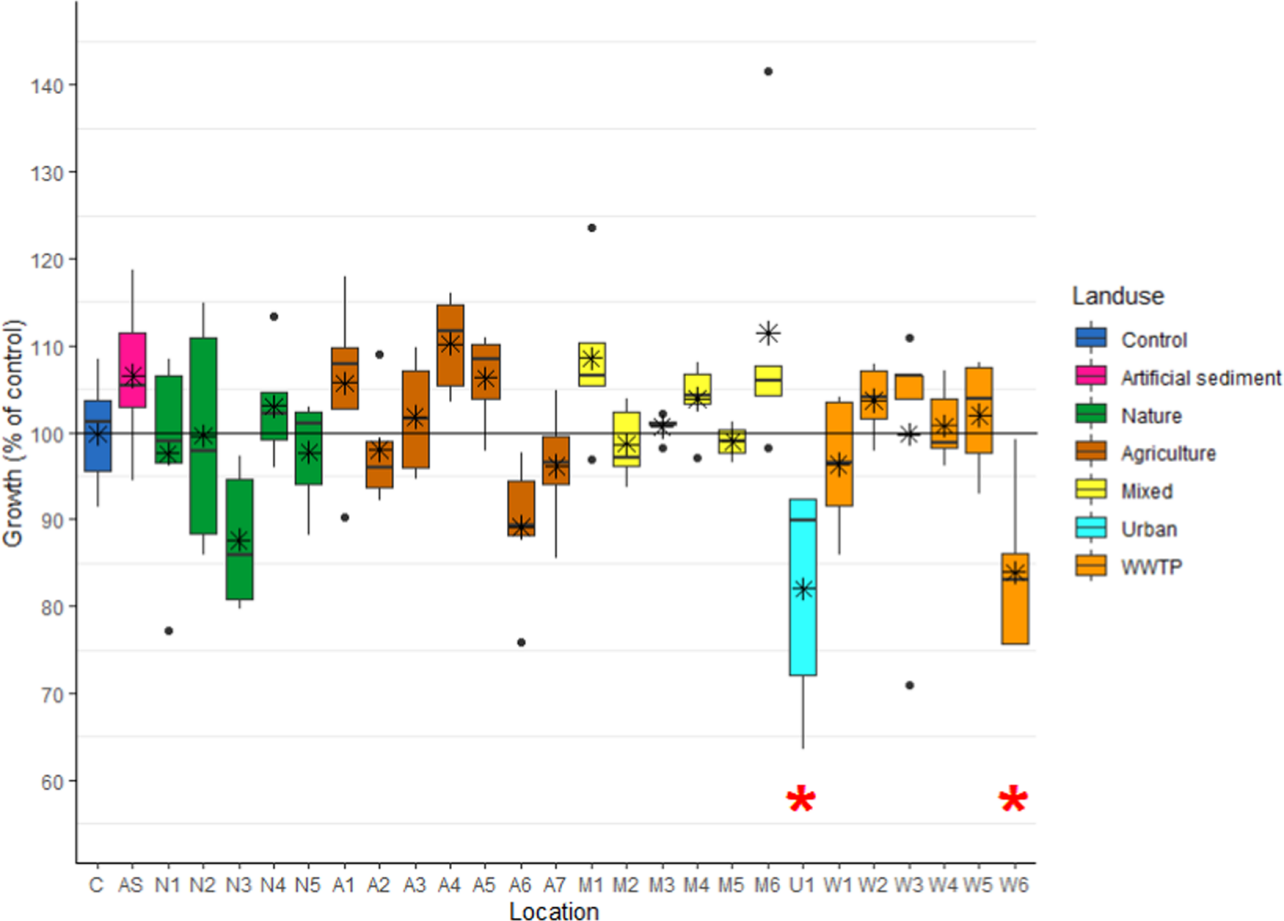
Growth of *C. riparius* larvae (% of control, *n* = 5) after 96 h of exposure to medium dosed by SR (*n* = 5) equilibrated with sediment originating from different locations (*n* = 25). Colors indicate land use: C = control, AS = artificial sediment, N = nature, A = agriculture, M = mixed, U = urban, and W = WWTP. The black line represents the average control growth, the black lines within the boxes represent the median growth per location, the black asterix within the boxes represents the mean growth per location, dots indicate outliers, a red asterix indicates significant (*p* < 0.05) differences in growth compared to the control.

In contrast to survival, growth of *C. riparius* larvae was significantly (*p* < 0.05) lower after exposure to the bioavailable fraction of U1 sediment (82.5 ± 3.4%, *p* < 0.01) and W6 sediment (83.9 ± 2.8%, *p* < 0.05) compared to the control (100 ± 2.1%, 0.35 ± 0.01 mm) (Figure 5). Hence, two of the 25 sediments affected larval growth.

## 4 Discussion

### 4.1 Experimental Considerations

This first attempt to demonstrate that SR can reliably transfer the chemical activity (i.e., bioavailable concentration) of a hydrophobic contaminant from the pore water of the sediment into an aqueous test medium using independent SPME measurements to validate this process was successful. As this study was conducted with a single chemical, follow-up research with other chemicals is needed to confirm that this promising method works for a wider spectrum of log *K*_OW_s. The developed method could be further extended by measuring the freely dissolved concentrations in the actual bioassay to confirm that the exposure concentrations are maintained during the 96 h test period and by performing whole-sediment toxicity tests to confirm results of the PSPD test.

Other approaches for diagnosing the cause of sediment ecotoxicity may be practically applicable, such as combining a whole-sediment test and sediment–water interface test for test organisms that ingest sediment particles^69^ or combining toxicity identification evaluation (TIE) and effect-directed analysis (EDA).^70^

### 4.2 SR as Rapid and Reliable PS and PD Material

Polymers like SR are commonly used as passive samplers to take up hydrophobic substances from water, air, and sediments.^49,50^ Equilibrium partitioning between water and sampler as well as between pore water and sampler increases with decreasing log *K*_OW_ of the compound.^51^ Sampling kinetics depend on the ratio between the sampler surface and thickness on the one hand and the volume of water on the other. For the passive dosing kinetics, the critical parameter is the ratio between the polymer surface and the volume of the receiving medium.^52^ The log *K*_OW_ values of the seven pesticides and the two PAHs ranged from 1.5 to 5.0, and swift equilibrium partitioning for the 0.5 mm thick SR material was demonstrated within several days. The presently obtained equilibrium times were on the short end of those reported, 1–20 days for different PAHs^34,53^ and ∼7 days for pesticides.^54^ Compounds that are more hydrophobic (log *K*_OW_ > 6) will take more time to equilibrate, and will equilibrate with the SR after 2–4 weeks with most organic sediment contaminants of ecotoxicological concern.^26,51^ The thickness of the SR is helpful in realizing a suitable passive doser volume with a limited surface area, allowing to effectively dose small aquatic volumes, as well as to facilitate chemical analysis of contaminants accumulated in the polymer.

For pesticides with a log *K*_OW_ < 2, the active substance is considered to be poorly sorbed to the sediment. As was clearly shown, the SR could also accumulate and release these more polar pesticides to some extent, although the yield was lower and depletion from SR in the PD may occur (see depletion calculation below).

While SR has previously proved its reliability as passive samplers, its subsequent use as a passive dosing material has been less well explored. Gilbert et al.^55^ showed that SR can be used as a passive doser to transfer *in vivo* exposure in humans (silicone implants) to *in vitro* assays (partition-controlled dosing). Smith and Jeong^56^ suggested that by combining equilibrium passive sampling and dosing, the bioavailable mixture profile can be transferred from an environmental sample into toxicity tests. Hence, to examine if the PS employed in the present study was able to serve as passive dosing devices in bioassays, the chlorpyrifos concentrations released from the SR into an aqueous test medium were compared with those in the pore water of the spiked sediment. To our knowledge, this is the first attempt to demonstrate that SR can reliably transfer the chemical activity (i.e., bioavailable concentration) of a hydrophobic contaminant from the pore water of the sediment into an aqueous test medium, using independent SPME measurements to validate this process. The next step would be to establish the silicone rubber–water partition coefficients for typical sediment contaminants in order to relate the concentrations in the silicone material to the freely dissolved concentrations.

The results of this experiment confirmed that SR can indeed serve as an efficient passive dosing device to deliver the accumulated contaminants into an aqueous phase and hence in a bioassay. The ratios of EqP between sediment and pore water predicted to observed dissolved concentrations as well as the partition coefficient of SR to polyacrylate (*K*_SR-PA_) are expected to be approximately constant and independent of the contaminant concentration in the sediment. However, the measured freely dissolved concentrations predicted using EqP were higher than observed, and the predicted to measured ratio increased as the sediment spiking concentration decreased. Also, the apparent silicone rubber to polyacrylate partition coefficient increased as the sediment spiking concentration decreased. This may be explained by concentration-dependent degradation of chlorpyrifos, which apparently influenced the dissolved concentrations similarly in spiked sediment pore water and in PSPD test media.

### 4.3 PSPD Bioassay with *C. riparius* Larvae

Equilibrium-based passive samplers absorb and redeliver the bioavailable contaminants in accordance with the partition ratios of these contaminants. As a prerequisite for achieving an undisturbed equilibrium, the volume of the SR should not deplete the sediment-sorbed concentration by >20%, hence a maximum of 1 g of SR per 10 g sediment organic matter should be applied. Likewise, also the aqueous test medium should not deplete the SR concentration by >20%. By applying the current PD bioassay with 100 mg of SR in 2 mL of medium with 0.175 mg of food, this would render 17% depletion of an organic contaminant with a log *K*_OW_ of 2 (Figures S3 and S4). Hence, in the present study, undisturbed equilibrium was achieved, both in the PS phase and in the PD phase.

By using polymer passive samplers as passive dosers, the absorbed contaminants are re-established into an aqueous solution, where they remain at a constant level.^57^ Using this principle, different passive dosing methods have been employed in bioassays where SR was loaded with single PAHs or recreated PAH mixtures,^35,56^ or was loaded with the influent and effluent of a wastewater treatment plant,^56^ after which the SR samplers were successfully used as dosers.^35,56^ Building on those findings, this study is the first one to subject contaminated field sediments to a newly developed PSPD test.

By employing SR, we managed to transfer the freely dissolved hydrophobic organic contaminant concentration from sediment pore water into an aqueous bioassay. Spiking sediment with different concentrations of the insecticide chlorpyrifos and employing these sediments to a PSPD test resulted in a 96 h water-only LC_50_ value for chironomid larvae of 22 ng/L, lower than reported in previous studies using chironomids, where LC_50_ values ranged from 70 to 825 ng/L (refs (35−41)). However, these previous studies used third or fourth instar larvae, while in the present study, the more sensitive first instar larvae were used. The higher sensitivity of the chironomid larvae in the present study can thus be explained by the life stage specific sensitivities of the chironomid larvae.^58^

Different *in vitro* bioassays have been used in previous PSPD research, such as Microtox and ER-Calux^56^ as well as different *in vivo* test organisms, like microalgae^35^ and amphipods,^59^ but *C. riparius* not yet. The results of the present study showed that first instar larvae of *C. riparius* seem to be a reliable test organism for PSPD evaluation of contaminated sediment as control survival, in both chlorpyrifos-spiked and field sediments, was high and growth of *C. riparius* larvae was significantly lower at two of the 25 field sediments. Although many confounding factors present in the sediment phase that influence the response of the test organism to chemical contaminants have been circumvented in the PSPD approach, *C. riparius* may still lack specific sensitivity to certain components of the chemical mixture that has been transferred via SR, in comparison to other animal test organisms and plants. Therefore, expanding the number of test species in the PSPD approach, preferably with reliable sublethal end points, will further reduce over- or underestimation of the risks of contaminated sediments.^13^ Possible test species include the isopod *Asellus aquaticus* and the daphnid *Daphia magna*, although the latter is not a sediment dweller.

### 4.4 Unraveling the Contribution of Hydrophobic Organic Contaminants to Sediment Ecotoxicity

Sediments are frequently the most contaminated environmental compartment,^2^ harboring complex mixtures of (un)known contaminants.^60−63^ The high cost management decisions on large-scale remediation, removal, relocation, and incineration of contaminated sediment should be well informed by adequate risk assessment. To isolate a specific part of the chemical stressors, the presently developed PSPD test sampled hydrophobic organic toxicants from a series of contaminated sediments which were dosed to an aqueous bioassay with larvae of the nonbiting midge *C. riparius*. Two out of 25 field sediments impacted larval growth, meaning that at these two locations, the concentration of the isolated hydrophobic organic contaminant mixture was high enough to affect insect larval growth, regardless of all of the other (non)chemical stressors present.

The next step would be to isolate the other stressor categories present in the contaminated sediments. To this end, other types of passive samplers can be deployed. For inorganic toxicants, such as metals, passive sampling can be achieved by means of diffusive gradient in thin films (DGTs);^36,64^ for polar organic compounds, such as pharmaceuticals, polyacrylate-coated solid-phase microextraction (SPME) fibers^36^ or polar organic chemical integrative samplers (POCISs) can be deployed;^65^ for amphiphilic compounds, such as PFAS, polycarbonate membrane (PC) can be used;^66^ and for organic cations, ion-exchange polymer can be used.^67,68^ However, to our knowledge, these passive samplers have not been used as passive dosing devices, as the extraction mechanism is based either on nonequilibrium adsorption (POCIS) or on complexation processes (DGTs), and sorption mechanisms are strongly affected by aqueous chemistry (PC). This makes these passive sampling materials less suitable for passive dosing applications.

It should be realized that, besides chemical contamination, aquatic ecosystems suffer from a wide variety of other stressors such as increased temperatures and droughts due to climate change, elevated turbidity, and nutrient concentrations due to eutrophication and habitat deterioration. Nonetheless, the current study provides a promising building block for a suite of simplified PSPD tests that after further validation, could be used to unravel the contribution of hydrophobic organic contaminants to sediment ecotoxicity, potentially affecting aquatic ecosystem health.

## Supporting Information Available

Additional information on the sampling locations; chemical target analyses; experimental information; and bioassay results (PDF)
