# Supplementary material for: Combining Passive Sampling and Dosing to Unravel the Contribution of Hydrophobic Organic Contaminants to Sediment Ecotoxicity
Source: Environ Sci Technol. 2023 Dec 28;58(1):269–79. doi: 10.1021/acs.est.3c07807 (PMC10785821; doi:10.1021/acs.est.3c07807)
Supplement: Supplementary file 1 — es3c07807_si_001.pdf [file es3c07807_si_001.pdf]

Supplementary Material

Combining passive sampling and dosing to unravel the contribution of hydrophobic organic contaminants to sediment ecotoxicity

Nienke Wieringa[1*], Steven T.J. Droge[1,2], Thomas L. Ter Laak[1,3], Aishwarya A.K. Nair[1], Kelsey Walker[1], Piet F.M. Verdonschot [1,2] & Michiel H.S. Kraak[1]

1. Department of Freshwater and Marine Ecology (FAME), Institute for Biodiversity and Ecosystem Dynamics (IBED), University of Amsterdam, Science Park 904, 1098 XH Amsterdam, The Netherlands
2. Wageningen Environmental Research, Wageningen University and Research, P.O. Box 47, 6700 AA Wageningen, The Netherlands
3. KWR Water Research Institute, Groningenhaven 7, 3433 PE Nieuwegein, The Netherlands

* Corresponding author: n.wieringa@uva.nl,

14 Pages

11 Tables

4 Figures

Table S1 : Table belonging to Figure 1 with additional information on the various components

	OC %	SR (g)	SPME	biota	sediment	water (mL)
**Passive sampling**						
Pesticide spiked sediment	3.1	2.2	-	-	800 g (wet)	300
PAH contaminated field sediment	4.7/9.3	2.2	-	-	500 g (wet)	500
**Passive dosing**						
Chlorpyrifos spiked sediment	3.1	2.8	-	-	750 g (dry)	750
Sediment sample with 3 SPME fibers	3.1	-	Yes	-	4 mL (wet)	4
Test medium with remaining SR strip and 3 SPME fibers	-	0.3	Yes	-	-	10
96 h water only bioassays with SR disc and *C. riparius*	-	0.1	-	Yes	-	1.8 (DSW)
**PS PD bioassays**						
Field collected sediment (25x)	Tabel S7	1.6	-		750 g (wet)	Added till 1 L
96 h water only bioassays with SR square and *C. riparius*	-	0.08	-	Yes	-	2 (DSW)

Table S1. Overview of the investigated study sites of the passive sampling experiment.

Location	Landuse	GPS	TOC (%)*
Science Park	Reference	52.356795, 4.955046	3.1
Bickersgracht	Urban	52.385376, 4.888233	9.3
Wittenkade	Urban	52.383030, 4.876830	4.7

* derived from previous research: De Baat ML, Wieringa N, Droge STJ, van Hal BG, Meer F Van Der, Kraak MHS. Smarter Sediment Screening: Effect-Based Quality Assessment, Chemical Pro fi ling, and Risk Identification. Environ Sci Technol. 2019;

Table S3. Overview of the seven pesticides used in the pesticide mixture sediment.

Chemical	CAS Number	Type	K_{oc}	Log K_{ow}	Water-sediment DT_{50} (d)[a] PPDB
**propoxur**	114-26-1	Insecticide	30	1.52	2
**pirimicarb**	23103-98-2	Insecticide	388	1.7	195
**carbofuran**	1563-66-2	Insecticide	25	2.32	9.7
**linuron**	330-55-2	Herbicide	341	3.2	24.1
**fipronil**	120068-37-3	Insecticide	749	4.0	68
**quinalphos**	13593-03-8	Insecticide	1465	4.44	21 (soil, not sediment)
**chlorpyrifos**	2921-88-2	Insecticide	9930	4.96	36.5

[a] data obtained from the Pesticide Properties Database (PPDB), see http://sitem.herts.ac.uk/aeru/ppdb/en/

Table S4. Concentrations of pesticides in the pesticide mixture sediment.

Chemical	Acetone spike (mg/L)	Volume of acetone spike (mL)	Weight of dry sediment spiked (g)	Sediment conc. (mg/kg dw)	OC conc. (mg/kg) for f_{OC} 0.05	Pore water conc. via K_{oc} (µg/L)	***Aquatic LC50 estimate*** **(µg/L)**
propoxur	0.85	254.2	236.4	0.92	18.4	613	38.1
pirimicarb	3.96			4.26	85.2	220	19.1
carbofuran	0.20			0.22	4.3	173	15.7
linuron	133.8			144	2878.6	8441	7150
fipronil	6.74			7.24	144.9	193	17.9
quinalphos	0.15			0.17	3.3	2.3	0.21
chlorpyrifos	5.45			5.86	117.2	11.8	0.05-0.16

Section S1. Chemical target analysis of sediment associated pesticides: QA/QC.

Separation and analysis were performed on a Shimadzu Prominence XR liquid chromatography system (LC), coupled to a Sciex 4000 QTRAP mass spectrometer (MS/MS, Applied Biosystems) with electrospray ionization (ESI). The LC was equipped with a binary LC-20AD XR gradient pumping system, SIL-20AC autosampler, CTO-20AC column oven, and CBM-20A controller. For the analysis, a C18 stationary phase (Acquity C18(2), 1.7 µm, 130 Å, 150 × 2.1 mm ID, Phenomenex) was used. For the quantification, transitions were measured in the positive ion mode with multiple reaction monitoring (MRM), see Table S4.

The positive mode MS method used initially 70% of solvent A (MQ-water + 0.5% acetic acid) and 30% B (acetonitrile) at a flow rate of 0.3 mL/min. After the first 5 minutes at this eluent composition, which should elute inorganic impurities that go to the waste for the first 3 minutes, but still retain the most polar pesticides. Eluent B was increased stepwise up to 100% within 15 minutes, kept at 100% for 10 minutes and then switched back to 30% B for the final 4 minutes (29 minutes total run time). In between the samples, the HPLC equilibrated for 7 minutes at 30% B.

The results of the LC-MS/MS analysis were gathered with the Analyst Software. The analyte concentrations in the samples were calculated from these results by using pre-made calibration standards. For each analyte, an external calibration curve was constructed using 10 mixed standards with known concentrations (333 – 0.017 µg L-1), with signals detected for the most abundant specific fragments from parent compounds. Using a weighting factor of 1/(peak area^2) on the orders of magnitude range of the detection signal, a linear curve was then used to calculate the analyte concentrations in the sample. The calibration curves were plotted using least-square regression. Each analyte showed sufficient linearity for the LC-MS/MS analysis in the studied working range (Limit of quantification set as the lowest point of the calibration curve with accuracy 80-120%), with correlation coefficients (R2) greater than 0.950. Subsequently, the measured concentrations in each sample were converted into measured concentrations per sampler in Microsoft Excel by accounting for dilution steps and extraction volumes.

Table S5. Compounds targeted in chemical profiling of the investigated sediments and their respective limits of quantification (LOQ) in positive mode.

Analyte Peak Name	CAS nr.	Analyte Retention Time (min)	Q1 (m/z)	Q3 (m/z)	LOQ injected (µg/L) [a]	LOQ for SR levels following the extraction protocol [b] (µg/kg)	Accuracy (%) lowest calibration sample [c]
carbofuran	1563-66-2	7.87	222.1	165.1	0.09	1.4	101%
chlorpyrifos	2921-88-2	10.4	349.9	97.0	3.46	51.9	96%
fipronil	120068-37-3	9.22	454	368	0.78	11.7	97%
linuron	330-55-2	8.77	249.1	160.1	0.12	1.8	105%
pirimicarb	23103-98-2	8.09	239	72	0.15	2.3	99%
propoxur	114-26-1	7.83	210	111	0.15	2.3	98%
quinalphos	13593-03-8	9.43	299	162.9	0.71	10.7	108%

[a] lowest point on a log-linear 5 level calibration curve

[b] using extraction of 0.1 g SR with 1.5 mL solvent, see Methods section in the manuscript

[c] back-calculated from the log-linear calibration curve and the nominal concentration.

Section S2. Chemical target analysis of sediment associated PAH: QA/QC.

Separation and analysis were performed on a Shimadzu Prominence XR liquid chromatography system (LC) coupled to a Prominence Fluorescence detector. The LC was equipped with a binary LC-20AD XR gradient pumping system, SIL-20AC autosampler, CTO-20AC column oven, and CBM-20A controller. The chemicals were separated on a LiChrospher C18 column (5µm; 250 x 4 mm; Knauer, Berlin, Germany). Phenanthrene fluorescence was measured at 250/385 nm and pyrene fluorescence was measured at 335/383 nm. PAH concentrations were quantified with external standard calibrations at a minimum of 5 levels and an accuracy of the log-linear calibration curve within 5%. Measurements started with a 45:55 acetonitrile:water (v:v) solution. For PAH detection in the acetonitrile SR extracts, after 1 minute, the acetonitrile concentration was slowly increased to 80% at 8 minutes. From minute 8 to 11, the acetonitrile concentration was increased to 100%, after which the concentration was decreased to 45% at 15 minutes.

Table S6. Compounds targeted in chemical profiling of the investigated sediments and their respective limits of quantification (LOQ).

Analyte Peak Name	CAS nr.	Analyte Retention Time (min)	fluorescence exc/em (nm)	LOQ injected (µg/L) [a]	LOQ for SR levels following the extraction protocol [b] (µg/kg)	Accuracy (%) lowest calibration sample [c]	log Kow (pub-chem)
phenanthrene	85-01-8	7.15	250/385	4.0	60	99%	4.46
pyrene	129-00-0	8.34	335/383	0.8	12	104%	4.88

[a] lowest point on a log-linear 5 level calibration curve

[b] using extraction of 0.1 g SR with 1.5 mL solvent, see Methods section in the manuscript

[c] back-calculated from the log-linear calibration curve and the nominal concentration

Table S7. Nominal concentrations for spiked sediments with chlorpyrifos.

	Sediment concentration spiked (mg/kg)	Pore water concentration (µg/L) calculated, based on K_{oc} = 9930 and foc = 0.05
Chlorpyrifos A	26.9	54.28
Chlorpyrifos B	5.4	10.87
Chlorpyrifos C	1.1	2.17
Chlorpyrifos D	0.2	0.43
Chlorpyrifos E	0.043	0.09

Table S8. Overview of the investigated study sites.

Code	Name	Landuse	GPS	TOC (%)
C	Controle			-
AS	Artificieel sediment			5
N1	Rottige Meenthe	Nature	52.822966, 5.907352	31.7
N2	Akmarijpsterpolder	Nature	53.00620, 5.81206	30.6
N3	Nieuwkoop	Nature	52.135603, 4.810454	33.2
N4	Het Merkske	Nature	51.428039, 4.779675	0.8
N5	Tongelreep	Nature	51.30290, 5.48619	2.8
A1	Breezand	Agriculture	52.8913777, 4.8263377	9.4
A2	Petten	Agriculture	52.764267, 4.681099	0.6
A3	Lisse	Agriculture	52.285328, 4.542945	1.6
A4	Noordwijkerhout 1	Agriculture	52.289785, 4.510474	1.2
A5	Noordwijkerhout 2	Agriculture	52.284805, 4.498523	0.1
A6	Westland	Agriculture	51.961299, 4.192689	0.5
A7	Sexbierum	Agriculture	53.227556, 5.497638	1.5
M1	Heerhugowaard	Mixed	52.675820, 4.833983	3.6
M2	Oude Leij	Mixed	51.565061, 4.986958	8.0
M3	Kleine Aa	Mixed	51.407561, 5.693623	16.0
M4	Boschloop	Mixed	51.297781, 5.605229	1.5
M5	Tungelrooyse beek	Mixed	51.230632, 5.625396	0.2
M6	Sciencepark	Mixed	52.356795, 4.955046	3.1
U1	Wittenkade	Urban	52.380458, 4.873499	31.7
W1	Mijdrecht	WWTP	52.212067, 4.886281	30.6
W2	Horstermeer	WWTP	52.255688, 5.087562	33.2
W3	Groot Heiveltse Beek	WWTP	51.512806, 4.849233	0.8
W4	Zandleij	WWTP	51.6023119, 5.0757993	2.8
W5	Biest-Houtakker	WWTP	51.504168, 5.172194	31.7
W6	Hilversum	WWTP	52.252807, 5.243704	30.6

Table S9. The equilibrium partitioning time for the uptake of organic compounds by silicon rubber from contaminated sediments

Chemical	Log K_{ow}	Log K_{oc}	SR uptake curve C_{max} (mg/kg)	SR uptake curve k_e (1/d)	SR uptake curve k_e 95% c.i.	SR uptake curve 95% eq. time (d)	SR average duplicate	nr of sampling points (1-144h)
**Spiked reference sediment**								
Propoxur	1.5	1.5	0.39	-		<0.5	109%	10
Pirimicarb	1.7	2.6	0.14	-		<0.5	111%	10
Carbofuran	2.3	1.4	0.061	-		<0.5	106%	10
Linuron	3.2	2.5	0.13	2.2	1.1 to 3.2	1.4	108%	10
Fipronil	4.0	2.9	0.035	6.8	3.8 to 9.7	0.4	114%	10
Quinalphos	4.4	3.2	0.073	2.9	2.1 to 3.8	1.0	105%	10
Chlorpyrifos	5.0	4.0	9.6	1.2	0.76 to 1.6	2.5	103%	10
**Contaminated urban sediment**								
Phenanthrene	4.5	4.0[a]	2.6	4.9	2.7 to 7.0	0.6	107%	9
Pyrene	4.9	4.6[a]	2.3	3.9	2.5 to 5.3	0.8	102%	9

[a] average K_{oc} for 8 soils as reported in:

De Jong, L. W., Moldrup, P., De Jong, H., Celis, R. (2008) Sorption and leaching of short-term–aged PAHs in eight European soils: link to physicochemical properties and leaching of dissolved organic carbon. Soil Science 173 (1), p 13-24, DOI: 10.1097/ss.0b013e31815aea32

Figure S1. Pesticide (propoxur, pirimicarb and carbofuron) concentrations in silicone rubbers (SR) (n = 2) after different contact times (d) with sediment spiked with pesticides. The fitted lines represent a first order exponential uptake curve.

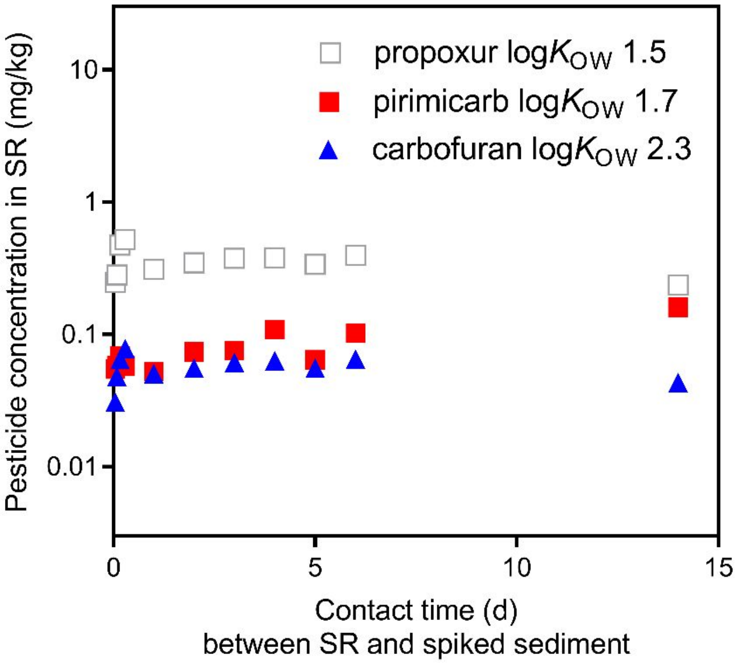

Table S10. Results of the 96 h PSPD bioassays with first instar larvae (<24 h) of the non-biting midge *C. riparius* with chlorpyrifos spiked sediment.

Sample	Acetone spike (mg/L)	Volume of acetone added (mL)	Total Sediment weight (g dw)	Sediment conc. (mg/kg dw)	SR conc. (mg/kg SR)	Average SPME conc. in sediment (mg/kg PA)	Average Dissolved conc. in PSPD via SPME (ng/L) * ± stdev	Average SPME conc. in PSPD (mg/kg PA)	Average Dissolved conc. in PSPD via SPME (ng/L) * ± stdev	Bioassay survival (%)
A	99.94	202	750	26.95	924.1	3848.4	34164 **(± 3239)**	5171.0	25426 **(± 565)**	0-0-0-0-0
B	19.99	200	740	5.40	190.4	456.4	3213 **(± 296)**	486.3	3015 **(± 162)**	0-0-0-0-0
C	4.00	200	741	1.08	42.7	109.9	587 **(± 69)**	88.9	725 **(± 26**	0-0-0-0-0
D	0.80	200	747	0.21	9.0	14.7	87 **(± 7.8)**	13.1	91 **(± 0.7, n=2)**	0-0-0-0-0
E	0.16	200	744	0.04	2.0	n.d.	15 **(± 1.7)**	2.3	11 **(± 0.56)**	80-100-100-100-100
Solvent control	0	200	740							100-100-100-100-100

***** *polyacrylate-water partition coefficient ($K_{PA\text{-}w}$) = 1.5 x 10^5 (log$K_{PA\text{-}w}$ = 5.18,* Magdic et al. 1996*) :*
Magdic S, Boyd-Boland A, Jinno K, Pawliszyn JB. Analysis of organophosphorus insecticides from environmental samples using solid-phase microextraction. J Chromatogr A. 1996;736(1–2):219–28.

Table S11a. The measured freely dissolved concentrations predicted using equilibrium partitioning (EqP). Ratio EqP predictions versus dissolved concentration in PSPD via SPME.

Sediment Concentration (Csed) (mg/kg)	EqP pore water prediction (μg/L)	Dissolved concentration in PSPD via SPME (μg/L)	Ratio
26.95	52.26	25.426	2.06
5.4	10.87	3.05	3.56
1.08	2.17	0.725	2.99
0.21	0.43	0.091	4.73
0.04	0.09	0.011	8.18

EqP pore water prediction = (Csed / foc) / Koc

Koc chlorpyrifos = 9930

Table S11b. Silicone rubber concentration to polyacrylate partition coefficient.

Sediment concentration (mg/kg)	SR concentration (mg/kg SR)	SPME concentration in PSPD (mg/kg PA)	$K_{SR\text{-}PA}$
26.95	924.1	5170	0.18
5.4	190.4	486.3	0.39
1.08	42.7	88.9	0.48
0.21	9.0	13.1	0.69
0.04	2.0	2.3	0.87

Figure S2. Survival of *C. riparius* larvae after 96 h of exposure to medium dosed by SR equilibrated (n = 5) with sediment originating from different locations (n = 25). Landuse: C = control, AS = artificial sediment, N = nature, A = agriculture, M = mixed, U = urban and W = WWTP.

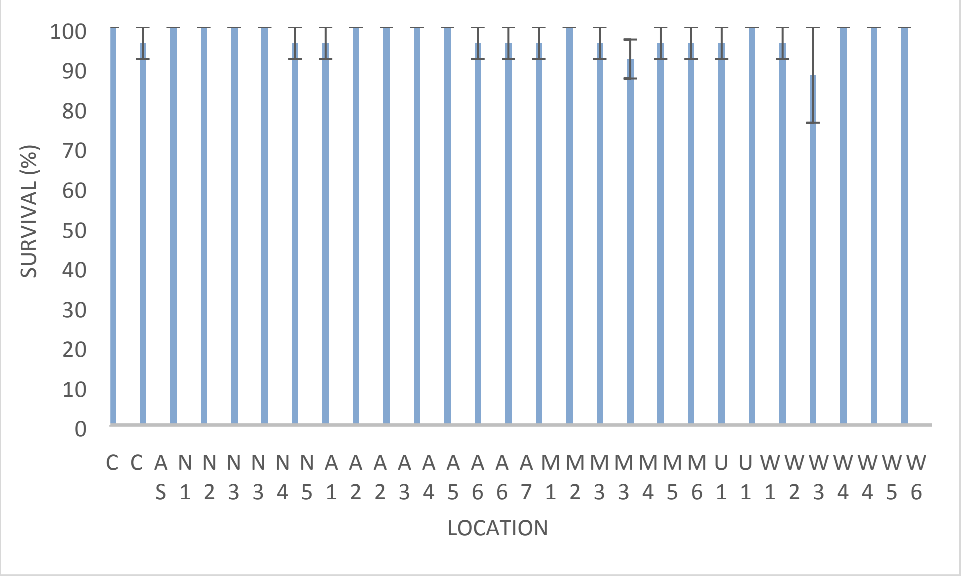

Figure S3. Calculations and assumptions on passive dosing depletion

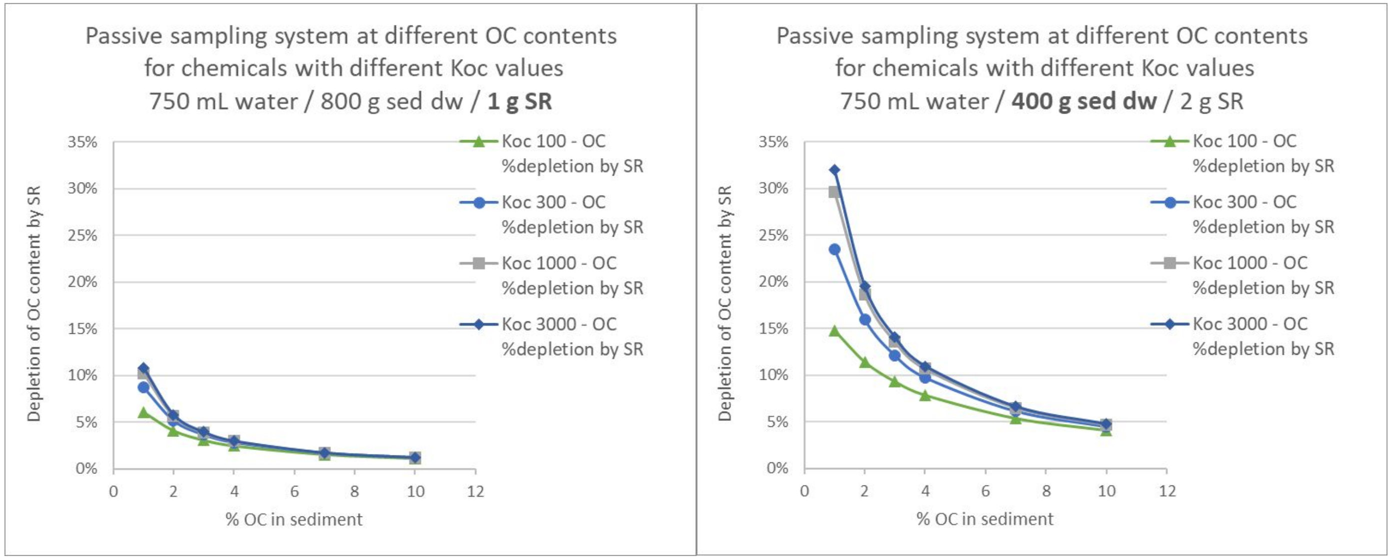

Assumptions:

Equation 1

$$M_{p(max)} = \frac{10^6 \times M_s \times f_{OC} \times K_{OC}}{K_{OC} \times \left(\frac{1}{0.2} - 1\right)}$$

Equation 2

$$K_{octanol-W} = K_{pW} = K_{OC} = K_{food-W}$$

Equation 1 derived from Jonker et al. 2020 with slight adjustment (0.2 instead of 0.05):

Jonker, M. T., Van Der Heijden, S. A., Adelman, D., Apell, J. N., Burgess, R. M., Choi, Y., ... & Wu, Y. (2018). Advancing the use of passive sampling in risk assessment and management of sediments contaminated with hydrophobic organic chemicals: Results of an international ex situ passive sampling interlaboratory comparison. *Environmental science & technology*, *52*(6), 3574-3582.

Figure S3 illustrates the influence of the OC content on the depletion of chemicals with different Koc, by the combinations 1g SR/800g sed (2g SR applied by us in 800 g sed) and 2g SR/400g sed, with a reference to the recommended equations in the Nature protocol on passive sampling by Jonker et al. (2020). With our applied settings, Here, we applied the threshold at maximum 20% depletion of OC-sorbed concentration, which would only be surpassed if 400 g or less sediment was used, for sediments with less than 2% OC, and at those limits it does not matter what the chemicals logK is (depending on the assumption of equal affinity for OC as for SR, which is well known to deviate to some extent).

Figure S4. Calculations and assumptions on passive sampling depletion. Note that both figures have identical data but Figure B applies a log scale Y axis to better display the chemical fractions predicted in food and larva.

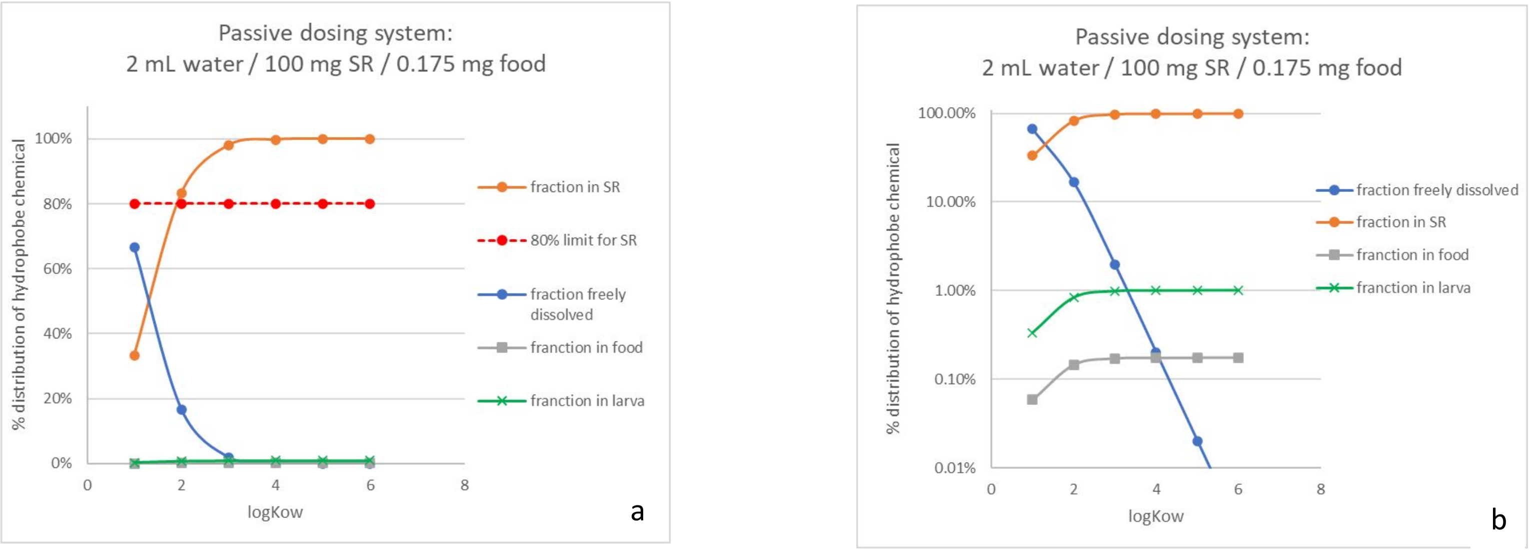

Figure 4 illustrates the depletion of chemicals from the passive dosing material volume/test medium volume/and food content, for chemicals with a different partition coefficients ranging log 1-6. Here, we applied the threshold at maximum 20% depletion of SR concentration, which would also only be limited for chemicals with logK < 2.
